# Rethinking antibiotics

**DOI:** 10.3389/fmicb.2015.00132

**Published:** 2015-02-24

**Authors:** Helen I. Zgurskaya

**Affiliations:** Department of Chemistry and Biochemistry, University of OklahomaNorman, OK, USA

**Keywords:** antibiotics, resistance, persisters, natural products, antimicrobial drug discovery and development

## Introductions

In the past 50 years, antibiotics became an important part of our everyday lives: we take them during routine visits to dental and doctor offices, but they are also invisibly present on supermarket produce shelves. If asked around, one would probably hear as many definitions of the word “antibiotic” as there are uses for these powerful drugs. However, this everyday presence was recently interrupted by more and more alarming news that these “miracle drugs” that saved millions of lives stopped working as expected. Antibiotic resistance became a matter of public news and executive orders of governments. The urgency of tackling antibiotic resistance requires actions from everyone. The new book “Antibiotics: Current Innovations and Future Trends” edited by Sergio Sanchez and Arnold L. Demain takes a fresh look at antibiotics as we know them and explores what we can do to preserve existing antibiotics and to secure the present and the future of modern medicine.

## What are antibiotics?

The book comprises 21 articles written by leading scientists on various aspects of antibiotic uses, activities, discovery and alternatives. Although the articles are unrelated, they are assembled in such a way as to truly engage a reader into the world of antibiotics. The starting article by Joan Wennstrom Bennett follows the history of defining “antibiotics” beginning with the “comprehensive” definition by Selman Waxman that an antibiotic is “… a chemical substance produced by micro-organisms… to inhibit the growth of and even to destroy bacteria and other microorganisms….” The subsequent chapters effectively demonstrate that the field of antibiotics has over-grown this definition and that medical uses of antibiotics include not only infectious diseases caused by micro-organisms but also oncological diseases (Chapter 2 by Ren and co-authors) as well as diseases caused by multicellular parasites (Chapter 9 by Shiomi and Omura) and viruses (Chapter 18 by Veiga-Crespo et al.). Non-medical uses are even more diverse and sometimes are troubling for example, feeding animals for growth, spraying flowers and fruits to preserve freshness and water treatments (Chapter 8 by Kardos). Although microorganisms remain to be major suppliers of antibiotics, present days these microorganisms come from new sources such as endophytes, geyzers and caves (Chapters 10–12). Even un-culturable micro-organisms are successfully “hunted” for new antibiotics (Lewis et al, Chapter 6). Animal venoms and plants also proved to be rich sources of peptides and small molecules with antibiotic properties (Samy et al., Chapter 13).

## How can we expand the diversity of antibiotics?

The empirical search for natural products in new and diverse places yielded most of the antibiotics that are currently used in medical and non-medical applications. This approach continues to expand the chemical diversity and biological activities of antibiotics. In addition, the book analyzes how more analytical approaches impact the drug discovery process and what we can expect in the future from these newest trends. Advances in DNA sequencing and genomics as well as in basic sciences impacted the drug discovery process in several major ways: (i) drug screening is re-focused from phenotypes onto specific targets (Chapter 14 by Lynn Silver); (ii) silent secondary metabolic pathways are identified from genome analyses and there are several ways to activate them (Chapter 15 by Martin and Liras); (iii) among various genome treasures are new enzymatic activities needed for combinatorial biosynthesis to generate novel bioactive compounds (Chapter 16 by Park and Yoon). Although some of these newest trends did not live up to high expectations—e.g., target focused screening was largely unproductive—they also suggested alternatives and solutions. Significant number of new antibiotics are currently in the development and clinical pipelines (Chapter 21 by Park and Thomas). Novel screening approaches focused on identification of pro-drugs effective against persisters, compounds acting on several homologous enzymes or pathways, efflux pump inhibitors, anti-virulence targets and pathogen specific vaccines all have a strong potential to expand the diversity and effectiveness of antibiotics.

## How can we preserve the existing activities?

The book does not specifically address the problem of resistance or mechanisms underlying the development and spread of antibiotic resistance. However, resistance affects and changes the entire field of antibiotics starting with the drug discovery process through the development and applications and is invariably present in almost all chapters. Undoubtedly, the misuse and overuse of antibiotics are major contributors to development and spread of resistance (Chapter 8 by Nelson Kardos). I would like to briefly re-cite for the readers the 2011 World Health Organization advises on interventions to address this growing problem: (i) lack of comprehensive national actions; (ii) lack of capacity and surveillance to choose adequate treatment for individual patients and make policy decisions; (iii) overuse of antibiotics for diseases that do not require them and underuse due to insufficient dosing and course durations; (iv) misuse of antibiotics in food-producing animals for growth promotion; (v) prevention of the spread of resistant bacteria in hospitals and their presence as environmental contaminants; (vi) inadequate momentum in research and development in the essential technologies. This book however demonstrates that we are gaining significant momentum in the essential technologies and many of these interventions are already in place. The major message is that only acting on a global basis, we can preserve antibiotics.

## Conclusions

This book would be of interest to a broad community of scientists, clinicians and administrators working with antibiotics. It could also be recommended as a textbook for upper level undergraduate and graduate classes at colleges and professional schools.

### Conflict of interest statement

The author declares that the research was conducted in the absence of any commercial or financial relationships that could be construed as a potential conflict of interest.

